# Transcatheter Aortic Valve Implantation Current Indications and Future Directions

**DOI:** 10.3389/fcvm.2019.00179

**Published:** 2019-12-18

**Authors:** Mirjam Gauri Winkel, Stefan Stortecky, Peter Wenaweser

**Affiliations:** ^1^Department of Cardiology, Inselspital, Bern University Hospital, University of Bern, Bern, Switzerland; ^2^Heart Clinic Hirslanden Zurich, Zurich, Switzerland

**Keywords:** valvular heart disease, aortic valve, transcatheter aortic valve replacement, structural interventions, aortic stenosis

## Abstract

Degenerative heart valve disease is associated with significant morbidity and mortality and healthcare expenditures. Transcatheter heart valve repair and replacement has introduced a fundamental change in the therapeutic management and transcatheter aortic valve replacement (TAVR) has gained substantial popularity. Favorable results from randomized trials and large real world registries lead to TAVR being considered a standard procedure with high rates of procedural success and low rates of peri-procedural complications. This article aims to review the past evolution, summarize the available evidence, discuss current indications and limitations and venture a glimpse into the future of percutaneous interventions for aortic valve disease.

## Introduction

Degenerative heart valve disease is frequently observed in the elderly and associated with a significant impact on patient morbidity and mortality (1), as well as healthcare expenditures (2). For decades, open-heart surgery was the only available therapeutic option for patients with significant valvular heart disease; nevertheless, more than 30% were denied appropriate treatment in the past (3). The advent of transcatheter heart valve repair and replacement has introduced a fundamental change in the therapeutic management of patients with significant heart valve disease. Since the first transcatheter treatment of symptomatic severe aortic stenosis in 2002 (4), transcatheter aortic valve replacement (TAVR) has gained substantial popularity, and favorable results from randomized trials and large real world registries supported the fast expansion of TAVR. During the past 17 years, a rapid adoption of TAVR was observed with increasing annual rates of TAVR procedures, whereas the ratio of observed vs. expected rates of mortality continuously declined (5). At this point in time, TAVR is established and considered a standard procedure with high rates of procedural success and low rates of peri-procedural complications. Conversely, transcatheter treatment options for significant mitral and tricuspid disease is still searching for comparable popularity and success.

This article will thoroughly review the past evolution of transcatheter aortic valve techniques, summarize the available evidence, discuss current indications and limitations and venture a glimpse into the future of percutaneous interventions for aortic valve disease.

## Aortic Valve Interventions

**Figure 1:**
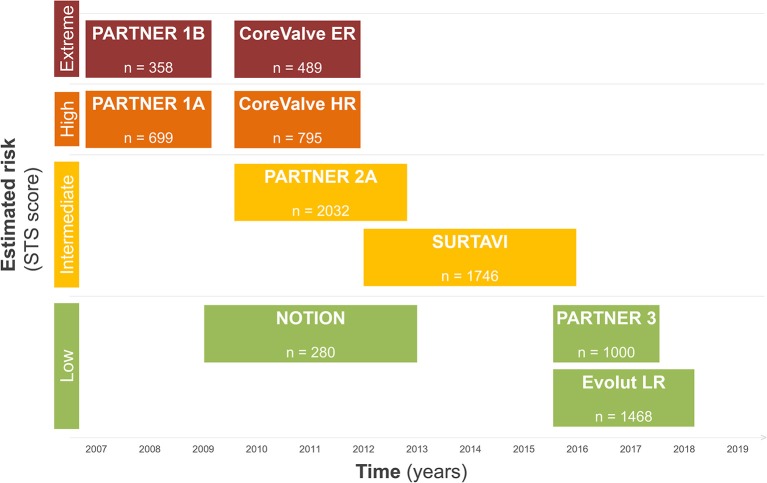
Overview of TAVR trials.

During the last decade, the treatment of degenerative aortic valve stenosis has undergone a tremendous evolution. Technological advances, procedural simplification as well as reproducible results in large observational registries and randomized trials has transformed the treatment of patients and resulted in a Class I guideline recommendation for use in symptomatic patients at increased surgical risk and in newer guidelines also in lower risk patients (6). Figure 1 summarizes the available evidence of TAVR according to surgical risk.

## The Evolution of TAVR—Evidence according to Surgical Risk

During the early experience, TAVR was exclusively reserved for patients considered to be inoperable or at prohibitive risk for surgical aortic valve replacement (7, 8). In this setting, the randomized controlled PARTNER [Placement of AoRTic TraNscathetER Valve] and the non-randomized CoreValve Extreme Risk trial were performed. While the PARTNER trial explored the effectiveness and safety of TAVR using the balloon-expandable Edwards Sapien transcatheter heart valve (THV) with standard medical treatment (PARTNER 1B), the CoreValve Extreme Risk trial used the self-expanding Medtronic CoreValve prosthesis in a prospective single-arm design. PARTNER 1B proved TAVR to be superior to medical therapy with an absolute 20% reduction in all-cause mortality at 1 year and a relative risk reduction of 50% at 5 years (9), while the CoreValve Extreme Risk trial corroborated the favorable PARTNER 1B result (10).

PARTNER 1A and the CoreValve U.S. Pivotal High Risk Trial were designed and powered to prove non-inferiority of TAVR vs. conventional surgical aortic valve replacement (SAVR) in selected high-risk patients (11, 12). While PARTNER 1A met the pre-specified criteria for non-inferiority for the primary endpoint of all-cause mortality and repeat hospitalization, the CoreValve High Risk Trial for the first time showed superiority for TAVR over SAVR for the primary endpoint death from any cause at 1 year follow-up. Based on the results of PARTNER 1B/1A and the CoreValve Extreme/High Risk trials the FDA approved TAVR in prohibitive risk and selected high-risk patients in 2011 and 2012 for the Edwards Sapien THV and in 2014 for the Medtronic CoreValve, respectively.

Over time, the risk profile of patients considered for a TAVR procedure progressively decreased, and results from trials in selected patients with intermediate surgical risk came to attention. The primary endpoint results from the randomized clinical PARTNER 2 trial were published in 2016 (13). TAVR using the Edwards Sapien XT prosthesis was found to be non-inferior to conventional surgery for the primary endpoint death and disabling stroke in intermediate risk patients; however, trialists found a higher rate of significant (≥moderate) paravalvular leakage with TAVR vs. SAVR. NOTION (Nordic Aortic Valve Intervention) was an all-comers trial investigating TAVR vs. SAVR and found lower rates of kidney injury, bleeding, and new-onset atrial fibrillation among patients undergoing TAVR (14). Similarly, also the SURTAVI (Surgical Replacement and Transcatheter Aortic Valve Implantation) trial using the self-expanding CoreValve or Evolut R prosthesis proved safety and efficacy of TAVR in intermediate surgical risk patients (15). Although surgical risk in TAVR patients continues to decline, patients remain elderly in the clinical trials of intermediate-risk patients, with a mean age of 79–82 years. Therefore, the results cannot necessarily be extrapolated to younger patients.

Low risk surgical patients were evaluated in randomized trials and observational studies and results of these studies were eagerly awaited, as they were considered to provide a turning point in the therapeutic approach for patients with severe aortic stenosis. In the PARTNER 3 trial, 1,000 patients with a mean Society of Surgeons risk score (STS) of 1.9% were randomly assigned to either TAVR or SAVR. Patients undergoing TAVR had lower rates of the composite endpoint including death, stroke and rehospitalization at 1-year follow-up (16). Furthermore, patients undergoing a TAVR procedure had a shorter hospital length of stay during the index hospitalization, a lower risk of new onset atrial fibrillation and a better functional status at 30 days. Similarly, the Evolut Low Risk trial (17), demonstrated excellent outcome data for TAVR in terms of procedural safety, valve performance and clinical outcome at 24 months. Moreover, the Low Risk TAVR (LRT) study (18), a prospective observational safety study mirroring a “real-world” experience, provided mortality rates as low as 0 and 3.0% at 30 days and 1 year, respectively. In this patient population, no disabling stroke and low rates of permanent pacemaker implantation (7.3%) were reported at 1 year follow-up. The results of these trials need to be cautiously interpreted, as all of them had relevant key exclusion criteria, such as concomitant significant coronary artery disease, bicuspid aortic valve anatomy, heavy calcification of the left ventricular outflow tract and pre-existing multi-valvular heart disease. An overview of the clinical trial results is highlighted in Table 1. A recently published meta-analysis encompassing more than 8,000 patients undergoing TAVR or SAVR from seven randomized controlled trials found that TAVR was associated with lower rates of all-cause mortality and stroke during 2 years of follow-up, independent of surgical risk (19). Furthermore, although procedural costs are higher, TAVR was found to be cost-effective when compared with SAVR. The significant difference was explained by lower overall costs of the index hospitalization, shorter length of hospital stay, and a higher quality-adjusted life expectancy for patients undergoing TAVR (20).

**Table 1:** Estimated and observed risk in TAVR vs. SAVR trials.

	**STS score (mean)**	**Mortality TAVR (30 days/1 year)**	**Mortality SAVR (30 days/1 year)**	**Ratio observed/expected**
PARTNER 1B	11.6 ± 6.0%	5.0/30.7%	–	0.43
CoreValve ER	10.3 ± 5.5%	8.4/24.3%	–	0.82
PARTNER 1A	11.8%	3.4/24.2%	6.5/26.8%	0.29
CoreValve HR	7.4%	3.3/14.2%	4.5/19.1%	0.45
PARTNER 2	5.8%	3.9/12.3%	4.1/12.9%	0.67
SURTAVI	4.5 ± 1.6%	2.2/6.7%	3.9/8.8%	0.49
NOTION	3.0 ± 1.7%	2.1/4.9%	3.7/7.5%	0.70
PARTNER 3	1.9%	0.4/1.0%	1.1/2.5%	0.21
Evolut LR	1.0 ± 0.7%	0.5/2.4%	1.3/3.0%	0.50

TAVR was rapidly adopted across all risk categories. By 2016, the less-invasive treatment was predominantly performed and patient numbers exceeded the proportion of patients undergoing isolated SAVR or combined SAVR and coronary artery bypass graft surgery (CABG) in Europe and the US (21). Indeed, the patient likelihood to undergo TAVR rather than SAVR has 4.6-fold increased between 2012 and 2016 (22). Increasing operator experience, technical improvement of different device iterations and a streamlined peri-procedural work-up process translated into a continuous improvement of device success and clinical outcomes during short- and longer-term follow-up. The cornerstone of today's treatment is a tailored approach for each patient, following an interdisciplinary discussion in the heart team. Anatomical characteristics and operator experience should determine the appropriate THV for the individual patient. Details of newer-generation transcatheter heart valve prostheses can be found in Table 2.

**Table 2:** Characteristics of newer-generation TAVR devices.

	**Deployment mechanism**	**Frame**	**Access**	**Skirt/Cuff/** **Seal**	**Anti-calcification treatment**	**Repositionable**	**Minimal sheath dimension (transfemoral)**	**CE mark**	**FDA approval**
Edwards Sapien 3	Balloon-expandable	Cobald chromium alloy	Transfemoral, transapical, transaortic	Yes (Polyethylen terephtalate)	Thermafix process™	No	14F	Yes	Yes (low/intermediate/high risk)
Medtronic Evolut R/Pro	Self-expanding	Nitinol	Transfemoral	No/Yes (Porcine Pericardium)	Alpha-amino Oleic Acid	Yes	14F	Yes	Yes (low/intermediate/high risk)
Boston Scientific Acurate Neo	Self-expanding	Nitinol	Transfemoral, transapical	Yes (Polyethylen terephtalate)	Biofix™	No	18F	Yes	No
Abbott Portico	Self-expanding	Nitinol	Transfemoral, transsubclavia, transaortic	Yes (Porcine)	Linx AC technology™	Yes	18F	Yes	No
NVT Allegra	Self-expanding	Nitinol	Transfemoral	No	No	Yes	18F	Yes	No
Boston Scientific Lotus Edge	Mechanically-expandable	Braided Nitinol	Transfemoral	Yes (Porcine Pericardium)	T-Guard™	Yes	18F	Yes	Yes (high risk)

## Treatment decision and current limitations

Over the last 17 years, TAVR has evolved from a procedure in well-selected very-high risk patients to a first choice treatment for the majority of patients with symptomatic severe aortic stenosis, whereas surgical treatment will remain to be discussed, including the following clinical scenarios or anatomical specifications:

**Coronary artery disease (CAD)** is the most common comorbid condition in patients eligible for TAVR. Owing to the continuous decline of surgical risk profile over time, a significant decrease in CAD prevalence was observed (23). Nevertheless, careful attention should be given to the severity of CAD during the pre-evaluation and the screening for a TAVR procedure, as the complexity of CAD was independently associated with cardiac mortality during the first 12 months after TAVR (24). Moreover, incomplete revascularization as indicated by a higher residual Syntax score was associated with a higher rate of the composite of cardiovascular death, stroke, or myocardial infarction at 1 year follow-up. Interdisciplinary Heart Team discussion and treatment decision is required for patients with significant CAD and severe aortic stenosis to identify the most appropriate treatment to effectively treat the aortic valve and provide full coronary revascularization, which is in line with current guideline recommendations (25–27). Moreover, the facility to access to the coronary ostia differs in the different TAVR valve constructions and can be either easy or a bit more complex after the valve implantation. This topic has become more and more important as younger patients are treated with TAVR and the possibility of a necessary PCI after TAVR increases. During the selection process for a specific TAVR prosthesis, this represents a key point in decision-making.

**Bicuspid aortic valve (BAV) anatomy** poses a challenge in the pre-procedural planning process for TAVR. Specific anatomic characteristics of bicuspid aortic valves may include an eccentric aortic annulus, asymmetric and excessive calcification, dilation of the aortic sinus and large diameters of the ascending aorta (28). A detailed and meticulous imaging assessment is required to fully appreciate and understand the anatomical specifications of the BAV anatomy. It is important to note that due to these anatomical challenges patients with bicuspid aortic valve anatomy have been systematically excluded from large randomized trials and only limited data exists on valve hemodynamics and clinical outcomes after TAVR in bicuspid anatomy. Early studies indicated low rates of peri-procedural device success and higher rates of conversion to surgery in patients with bicuspid valve anatomy, and relevant differences between early and newer generation TAVR devices were identified (29). Most recently, the STS/ACC Transcatheter Valve Therapies (TVT) Registry provided some in-depth insights into TAVR for BAV. In a propensity score matched patient cohort of almost 2,700 patients with bicuspid and tricuspid aortic valves, the STS/ACC TVT Registry provided similar rates of mortality between groups at 30 days, but higher rates of stroke among patients with BAV disease. Patients with BAV were at higher risk for conversion to open heart surgery, but had similar hemodynamic outcome, paravalvular aortic regurgitation and health related quality of life after successful TAVR at 12 months, when compared with tricuspid aortic valve patients (30, 31). While the results of this large patient cohort are reassuring, it remains to be elucidated, whether differences in BAV phenotype—according to raphe morphology (32) or the Sievers classification (31, 33)—may affect procedural success and clinical outcomes with TAVR. A detailed and meticulous imaging assessment is required to fully appreciate and understand the anatomical specifications of the BAV anatomy. In order to minimize the risk of annular rupture or conduction disturbances, sizing of THV in BAV should be conservative. In patients with BAV and concomitant disease and dilation of the ascending aorta a surgical replacement should be considered.

**Native aortic regurgitation (AR)** is still considered an anatomical contraindication for most of the available transcatheter heart valve devices and the off-label use of TAVR in this setting is not recommended (34). Concomitant dilation of the ascending aorta as well as large aortic annulus diameter are frequent coexisting anatomical characteristics of pure AR, challenging an appropriate device selection. Currently, the majority of available TAVR devices are designed for treating degenerative and calcified aortic valve anatomies, relying on the fixation and anchoring of the prosthesis within a calcified aortic annulus. The combination of missing anatomical landmarks during the procedure as well as incomplete fixation within the aortic annulus due to the absence of valve calcification might potentially result in misplacement or migration of the transcatheter heart valve. In addition, the hypercontractile state of the left ventricle due to the increased stroke volume with a dynamic regurgitant jet limits device control during valve positioning and release (35). However, in selected patients with AR, TAVR provides an effective treatment option. According to a systemic review encompassing 175 high-risk patients with native AR, TAVR particularly using second-generation devices, was associated with excellent clinical outcomes and device success (36). Furthermore, the STS/ACC TVT Registry suggests that highly selected patients with AR considered inappropriate candidates for surgery, do benefit from a TAVR procedure with second generation devices (37). At this point in time dedicated devices for treating pure AR are limited. By now, only the JenaValve transcatheter heart valve (JenaValve Technology) and the J-Valve system (JC Medical) have been designed to address the morphological challenges in patients with AR, by providing a dedicated anchoring system in the aortic annulus in the absence of calcification. First clinical experiences reported a high procedural and device success rate for the JenaValve, leading to a CE Mark in Europe for the treatment of AR (38–40). In comparison to standard newer generation transcatheter heart valves, dedicated devices lead to a higher procedural success. However, there seems to be no difference in mortality, stroke or residual AR (41).

**Valve-in-valve (ViV)** treatment for failing surgical bioprostheses has gained substantial popularity, and is considered a valid alternative to avoid redo SAVR in elderly patients (42). Previous analyses from the Valve-in-Valve International Data (VIVID) registry provided promising results after ViV treatment during the first year of follow-up (43). The PARTNER 2 registry corroborated these favorable results in high-risk patients and added substantial insights into long-term clinical data up to 3-years follow-up after ViV treatment using the balloon-expandable Edwards Sapien transcatheter heart valve (44). In this PARTNER 2 analysis, the results of selected high-risk patients undergoing ViV treatment were reported, and after 3 years of follow-up the investigators observed rates of all-cause mortality as high as 32.7%; a rate, which was comparable to outcomes after TAVR in native aortic stenosis (44.2%) and after SAVR (44.8%) in a comparable patient risk cohort (12). Patients undergoing ViV treatment had excellent and sustained hemodynamic valve performance and maintained improvement in functional status and health related quality of life. In contrast to the report from the VIVID registry, PARTNER 2 patients had similar outcomes in subgroup-analyses of different valve sizes and in analyses of patient prosthesis mismatch after ViV TAVR. However, it needs to be mentioned that patients with surgical valves smaller than 21 mm were excluded from this trial. The recently published outcome data from the CoreValve US Expanded Use Study corroborate the favorable ViV results from the PARTNER 2A registry. At 3 years transcatheter ViV performance was maintained with low rates of reintervention and an improvement of effective orifice area over time (45).

ViV TAVR interventions require a sophisticated pre-operative evaluation process as procedural success might be offset by procedural complications like coronary artery obstruction or significant patient prosthesis mismatch (PPM). In order to decrease the likelihood of PPM after ViV, TAVR in patients with small sized surgical bioprosthesis, interventional techniques of valve frame fracture can be employed in selected types of surgical valves. Recent data from a multicenter study indicate low rates of procedural complications and favorable hemodynamic after surgical valve fracture during ViV TAVR (46). In addition, manufacturers have designed dedicated surgical valves to facilitate ViV TAVR implantation while reducing the risk for PPM (47). Coronary artery obstruction by displacing the prosthetic heart valve leaflet toward the coronary artery ostia during TAVR prosthesis deployment is a potential life threatening complication in selected patients. Patients are at increased risk for coronary artery occlusion in case of coronary artery height below 10 mm, shallow width of the Sinus of Valsalva (<30 mm) and in selected patients with externally mounted leaflets or stentless surgical bioprosthesis. In patients considered to be at high-risk for coronary artery occlusion during the pre-procedural evaluation for TAVR, selective protection strategies and interventional techniques are employed. One of these techniques includes an intentional splitting of the native or the prosthetic heart valve leaflet—the Bioprosthetic aortic scallop intentional laceration to prevent iatrogenic coronary artery obstruction (BASILICA) (48). During BASILICA, the target aortic leaflet is separated by using radiofrequency energy directed by catheters and guidewires, thereby splitting the leaflet in two pieces to allow coronary flow through the open cells of the TAVR prosthesis. In a population of 30 selected patients at anticipated high risk for coronary obstruction, BASILICA was successfully performed with high rates of procedural success and by avoiding coronary artery obstruction (49). Most recently, *in-vitro* studies suggested a role for BASILICA in the prevention of transcatheter heart valve thrombosis (50), through improvement of hemodynamics in the sinus and the neo-sinus (51).

Antithrombotic management after TAVR remains a field of uncertainty and subject of ongoing studies. Based on expert consensus, guidelines recommendations include dual antiplatelet therapy (DAPT) early after TAVR to prevent device-related thromboembolic events, followed by life-long single antiplatelet therapy (6). The available literature, however suggests an increased risk of bleeding complications during DAPT. Indeed, while pretreatment with DAPT was an independent risk factor of in-hospital bleeding (52), DAPT after TAVR was consistently associated with bleeding during follow-up without providing a benefit for ischemic outcomes like myocardial infarction, stroke or mortality (53). Furthermore, while observational studies including sophisticated computed tomography imaging showed lower rates of subclinical leaflet thrombosis in patients receiving oral anticoagulation rather than DAPT (54), the randomized GALILEO trial was stopped prematurely as preliminary analyses showed risks of all-cause death and bleeding post-TAVR to be doubled among patients receiving Rivaroxaban rather than DAPT.

## The Future of TAVR

**Figure 2:**
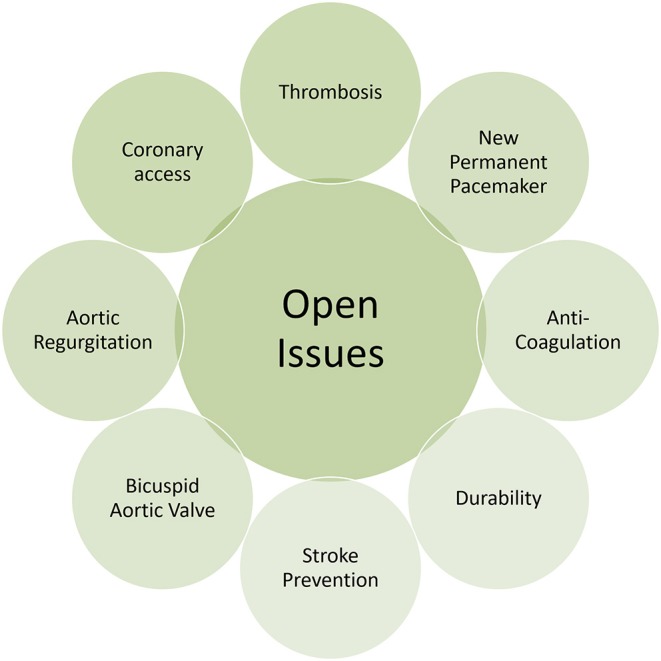
Issues to be addressed by future devices.

Based on the available literature and irrespective of clinical risk profile, it is likely that TAVR is at least non-inferior to SAVR when it comes to hard clinical endpoints like stroke or mortality. Moreover, recent data inform on superiority when TAVR can be delivered through the less-invasive femoral access route, which is employed in more than 92% of patients undergoing TAVR (5). Considering the favorable literature and the rapid adoption of TAVR in surgical low risk patients, it seems inevitable that this movement will go on and will include younger patients considered to receive an aortic bioprosthesis. Current limitations and drawbacks however need to be considered and resolved beforehand (Figure 2). When extending the indication for TAVR to patients with longer life expectancy, the issue of heart valve durability needs to be addressed. Currently, only limited evidence exists on heart valve durability and rates of structural valve deterioration (SVD) beyond 7 years of follow-up. Randomized trials and observational studies, however consistently proved favorable valve performance without significant structural valve deterioration during 5-years of follow up (9, 55–57). The NOTION trial added information on 6-years clinical follow-up and showed higher rates of SVD among patients undergoing SAVR than TAVR (24.0 vs. 4.8%), whereas heart valve failure, defined as valve-related death, aortic valve re-intervention or severe SVD, was similar in both groups (58). Repeat transcatheter heart valve intervention, meaning TAV-in-TAV, is a field in evolution and only some limited experience has been reported so far (59).

Novel designs of transcatheter heart valves as well as alterations of established valves keep on expediting the evolution of TAVR. New concepts, like the dry leaflet technology or dedicated motorized deployment mechanisms are evaluated for their application in clinical practice. Most recently, a glimpse into the future was provided from biomedical engineering with novel and innovative devices consisting of bio-polymeric heart valves. *In-vitro* testing has shown promising results (60–63), and it remains to be seen if, and when the technology can be implemented in clinical studies.

Whether the future of aortic valve treatment will be limited and restricted to patients with typical symptoms is going to be addressed in the EARLY TAVR trial (Evaluation of Transcatheter Aortic Valve Replacement Compared to SurveilLance for Patients With AsYmptomatic Severe Aortic Stenosis) (64). This ongoing trial seeks to investigate the value of TAVR in asymptomatic patients with severe aortic stenosis (64). While risk stratification might be challenging in this patient population, several factors have to be taken into account to appropriately identify patients (65). Left ventricular global strain (LV GLS) using echocardiography has been promoted as a tool to identify patients with global intact LV-function and severe aortic stenosis that would benefit from an early intervention. Impaired LV GLS is considered a marker for subclinical myocardial dysfunction that is often present in patients with asymptomatic severe AS with preserved LVEF. Over time and during the clinical course of aortic stenosis, LV GLS further deteriorates indicating the need for aortic valve intervention (66).

Whether guideline recommendations will be limited to patients with severe aortic stenosis in patients with pre-existing heart failure will be evaluated in the TAVR UNLOAD trial (Transcatheter Aortic Valve Replacement to UNload the Left ventricle in patients with ADvanced heart failure) (67, 68). In this trial, patients with symptomatic (≥NYHA II) impaired left ventricular function (LVEF < 50%) and moderate aortic stenosis will be randomized to a wait-and-see strategy with optimal medical therapy (OMT) or OMT plus TAVR (68). The study is based on the hypothesis that TAVR, by reducing the volume overload of the left ventricle, partially caused by the aortic stenosis, leads to a better outcome in patients with advanced heart failure with reduced ejection fraction.

## Summary

During the last decade, TAVR techniques and technology have continuously improved, making the procedure a safe and effective treatment for most of the patients. TAVR will continue to gain significant popularity among patients with aortic stenosis and it is expected that patient volume will continue to grow exponentially. In the near future, TAVR will become the treatment of choice for patients with single aortic stenosis and SAVR may be considered a complementary alternative for patients who are not ideal candidates for TAVR.

## Author Contributions

All authors have made substantial contributions to the conception of the work. It has been drafted by MW and SS and has been critically revised by all authors for important intellectual content. All authors have given their approval for publication of the content and have agreed to be accountable for all aspects of the work in ensuring that questions related to the accuracy or integrity of any part of the work are appropriately investigated and resolved.

### Conflict of Interest

PW declares proctor/lecture fees from Edwards Lifesciences and Medtronic. The remaining authors declare that the research was conducted in the absence of any commercial or financial relationships that could be construed as a potential conflict of interest.
